# Evaluation of the effectiveness of topical oily solution containing frankincense extract in the treatment of knee osteoarthritis: a randomized, double-blind, placebo-controlled clinical trial

**DOI:** 10.1186/s13104-023-06291-5

**Published:** 2023-03-04

**Authors:** Afsaneh Mohsenzadeh, Mansoor Karimifar, Rasool Soltani, Valiollah Hajhashemi

**Affiliations:** 1 Students Research Committee, School of Pharmacy and Pharmaceutical Sciences, Isfahan University of Medical Sciences, Isfahan, Iran; 2 Department of Rheumatology, School of Medicine, Isfahan University of Medical Sciences, Isfahan, Iran; 3 Department of Clinical Pharmacy and Pharmacy Practice, School of Pharmacy and Pharmaceutical Sciences, Isfahan University of Medical Sciences, Isfahan, Iran; 4 Infectious Diseases and Tropical Medicine Research Center, Isfahan University of Medical Sciences, Isfahan, Iran; 5 Department of Pharmacology and Toxicology, School of Pharmacy and Pharmaceutical Sciences, Isfahan University of Medical Sciences, Isfahan, Iran

**Keywords:** Knee Osteoarthritis, *Boswellia serrata*, Boswellic acid, Clinical trial

## Abstract

**Objective:**

Pharmacological treatments of osteoarthritis (OA) have several side effects. *Boswellia serrata* resin (frankincense) is rich in boswellic acids that have antioxidant and anti-inflammatory effects; though, their oral bioavailability is low. The aim of this study was evaluation of the clinical effectiveness of frankincense extract in the treatment of knee OA. In a randomized double-blind placebo-controlled clinical trial, eligible patients with knee OA were randomly divided into two groups of drug (33 patients) and control (37 patients), to use oily solution of frankincense extract or placebo, respectively, on the involved knee three times daily for four weeks. WOMAC (Western Ontario and McMaster Universities Osteoarthritis Index), VAS (visual analogue scale; for pain severity), and PGA (patient global assessment) scores were determined before and after intervention.

**Results:**

For all evaluated outcome variables, there was a significant decrease from baseline in both groups (P < 0.001 for all). Furthermore, the end-of-intervention values for all parameters were significantly lower in drug group than placebo group (P < 0.001 for all), showing more effectiveness of drug compared to placebo.

**Conclusion:**

Topical oily solution containing enriched extract of boswellic acids could decrease pain severity and improve the function in patients with knee OA.

*Trial Registration* Trial registration number: IRCT20150721023282N14. Trial registration date: September 20, 2020. The study was retrospectively registered in Iranian Registry of Clinical Trials (IRCT).

## Introduction

Osteoarthritis (OA) is one of the most common degenerative articular disorders causing negative impacts on the quality of patient’s life [1, 2]. Synovial inflammation, ligaments destruction and limitation of joint ambulation occur in OA [3, 4]. About 40% of patients aged 70 years or over are involved with knee OA with the proportion of women being more than men [5, 6]. So, age has an important role in OA incidence as well as obesity, genetics, history of knee trauma and female gender [7].

Compared to other kinds, knee OA is the most common, involving about 85% of cases [5, 6]. The patients may experience pain and instability of joint, morning stiffness, crepitus, and reduction of daily physical activity [8]. Exercise, weight loss, and patient’s education are the mainstay of OA treatment [9]. Based on the guidelines, the pharmacotherapy for OA consists of acetaminophen, NSAIDs (non-steroidal anti-inflammatory drugs), opioids, and intra-articular injection of glucocorticoids and hyaluronic acid. Although these drugs can improve quality of patient’s life and performance, knee pain and joint stiffness remain in some patients [10]. On the other hand, many adverse effects have been observed with some of the mentioned drugs, including renal and cardiovascular complications and gastrointestinal bleeding related to NSAIDs consumption [4].

*Boswellia serrata* is a plant from family Burseraceae with anti-inflammatory and antioxidant effects [2]. The extract of frankincense (the hardened gum-like resin obtained from Boswellia and also known as “Kondor” in Iran) is rich in boswellic acids, specially 3-O-acetyl-11-keto-β-boswellic acid (AKBA), which can inhibit 5-lipoxigenase enzyme as well as NF-ĸB (nuclear factor kappa B) and TNF-α (tumor necrosis factor α) in many inflammatory pathways [11, 12]. Due to these effects, some *in-vivo* and clinical studies have shown that oral consumption of Boswellia extract can reduce pain, swelling, and stiffness of joint in OA compared to standard treatment. However, poor absorption in aqueous intestinal environment and high first pass metabolism result in low bioavailability of boswellic acids during oral consumption [13]. Besides, in chronic inflammatory diseases, patients are usually more compliant for using topical treatments as an easier way with less side effects and drug interactions [14]. Although there is no clinically significant change in laboratory parameters due to oral consumption of Boswellia, several minor side effects have been recorded [15, 16]. On the contrary, there is no study to show the effects of topical Boswellia extract on knee OA. So, this study aimed to evaluate the potential effectiveness of topical extract of *B. serrata* in relieving the symptoms in patients with knee OA.

## Materials and methods

This was a randomized, double-blind, placebo-controlled clinical trial performed in Rheumatology Clinic of Al-Zahra hospital of Isfahan, Iran, affiliated to Isfahan University of Medical Sciences, from October 2020 to April 2021. The study was registered in Iranian Registry of Clinical Trials (IRCT) with the record number of IRCT20150721023282N14.

### Preparation of oily solutions

Topical formulation of frankincense extract was prepared by Fardis pharmaceutical company, Isfahan, Iran. For preparing 100 ml of Boswellia solution, 1 g of dried extract of frankincense was added to 20 ml of black seed oil, then reached to the volume with olive oil. The placebo solution was prepared just with 20 ml of black seed oil and 80 ml of olive oil. Both types of solution were similarly packaged in pharmaceutical tubes and labeled and a numeric code was recorded on each tube by the company.

### Patient selection.

Patients were selected from those referring to rheumatology clinic of Al-Zahra hospital, based on the following inclusion criteria: (1) age of 40–80 years, (2) OA of at least one knee for at least 3 months based on the diagnostic criteria of American College of Rheumatology (ACR), (3) pain score > 4 based on Visual Analogue Scale (VAS), and (4) grade 2 or 3 of Kellgren-Lawrence scale in knee radiography within the past 3 month.

The patients’ exclusion criteria were: (1) use of intra-articular glucocorticoids within the past 3 months, (2) use of intra-articular sodium hyaluronate within the past 6 months, (3) use of systemic glucocorticoids (either oral or parenteral) within the past 14 days, (4) concurrent other osteoarticular disorders (e.g., rheumatoid arthritis and gout), (5) any skin disorder in the knee region, (6) any allergic reaction to the prescribed topical preparation, (7) knee arthroscopy within the past 3 months, (8) illiteracy, (9) inability to answer the questions, and (13) pregnancy or lactation (for women).

### Clinical study and interventions.

All included participants filled a written informed consent form. Demographic and clinical characteristics of patients including age, gender, the comorbidities, and current consumed drugs were recorded for all patients. Before any intervention, the pain severity based on VAS (0 to 10 scale), and the scores of WOMAC (Western Ontario and McMaster Universities Osteoarthritis Index) and PGA (patient global assessment) were determined and recorded for the patients according to the symptoms experienced within the past 48 h. WOMAC index consists of three subscales including pain (5 questions), the flexibility of knee joint (2 questions), and the function or daily activities (17 questions) that a score range of every question presents the severity of the symptom. The total score of WOMAC ranges from 0 to 68, as the best and worst state for knee OA, respectively. The Persian form of WOMAC used in this study, has been validated previously by *Nadrian *et al. [17]*.* PGA is a visual measurement scale in the form of a colored band ranging from white zone/point 0 (without any symptoms) to black zone/point 4 (the worst symptoms) and it was marked based on patient self-assessment. Simple randomization method was used for random allocation. For this, the tubes containing drug or placebo were equally and randomly given to eligible patients and the numeric code of each tube was written on the patient’s data collection form. Patients were asked to apply the solution on the involved knee, three times daily for 4 weeks. Also, acetaminophen 500 mg three times a day was prescribed for all patients, as a standard treatment for OA. Furthermore, the patients were asked not to use any other drug/supplement for OA, including glucosamine, chondroitin, herbal medicines, and topical preparations, during the study period. The patients were instructed to record regular use of the solution and report any side effect during the study. As the tubes of drug and placebo solutions were fully similar, the prescribing physician (rheumatologist), the data collector, and the data analyst were all blinded to the type of intervention (drug vs. placebo) for each patient. At the end of the study, VAS, WOMAC, and PGA scores were recorded again for all patients. The type of intervention for each patient was decoded after data analysis.

The primary outcome measures were the changes of VAS, WOMAC, and PGA scores at the end of study. The secondary outcome variable was the rate of possible side effects based on the patients’ report.

### Sample size calculation.

The following equation was used for sample size calculation:

$${\text{n = }}{{\left( {{\text{Z}}_{{{1} - \alpha /{2}}} + {\text{ Z}}_{{{1} - \beta }} } \right)^{2} \times {2}\delta^{{2}} } \mathord{\left/ {\vphantom {{\left( {{\text{Z}}_{{{1} - \alpha /{2}}} + {\text{ Z}}_{{{1} - \beta }} } \right)^{2} \times {2}\delta^{{2}} } {\left( {\mu_{{1}} - \, \mu_{{2}} } \right)^{{2}} }}} \right. \kern-0pt} {\left( {\mu_{{1}} - \, \mu_{{2}} } \right)^{{2}} }}$$

where n is the required sample size in each group; µ_1_ and µ_2_ are the mean of the variable in the first and second groups, respectively, according to the previous studies; δ is the standard deviation (SD); Z_1-α/2_ is the standard normal z-value for a significance level α = 0.05, which is 1.96, and Z_1-β_ is the standard normal z-value for the power of 80%, which is 0.84. According to the µ and δ values for pain scores (in WOMAC) in a previous report [18], at least 25 patients were considered for each group.

### Statistical analysis

Data analysis was performed by SPSS 24.0 software (SPSS Inc., Chicago, IL, USA). Qualitative variables were compared by Chi square (χ2) test and Fishers’ exact test between the two groups. Kolmogorov–Smirnov test determined distribution pattern of quantitative data. The normally and non-normally distributed data were presented as mean (SD) and median [IQR], respectively. Wilcoxon Signed Rank test was applied for comparison of values at the beginning and end of intervention within each group. Mann-Whithney U test was applied for comparison of baseline values between the two groups. ANCOVA test was performed to compare the post-intervention values between the groups with the control of baseline values as covariates. *P*-value < 0.05 was considered as statistically significant.

## Results

### Patients

**Fig. 1:**
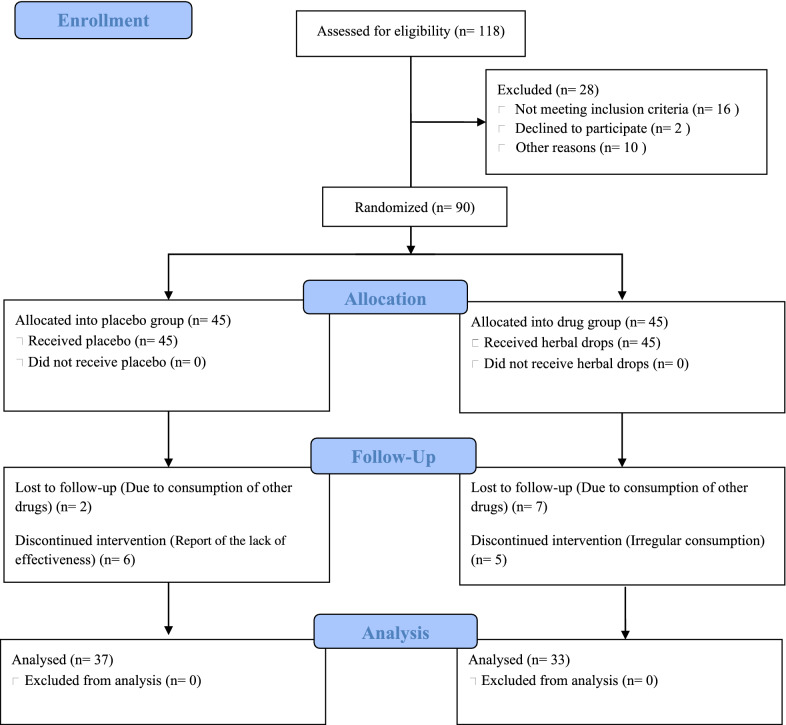
Flowchart of enrollment and allocation of participants and study design

During the study, 90 patients who met the inclusion criteria participated in the study. The patients aged between 41 to 77 years and were divided into two groups of drug and placebo. Because of irregular consumption or usage of similar products, 20 patients were excluded from the study. So, 70 patients completed the research including 33 and 37 patients in drug and placebo groups, respectively (Fig. 1). As shown in Table 1, there was no significant difference between the groups regarding baseline demographic and clinical characteristics, including age, gender, comorbidities, and concurrent drugs.

**Table 1:** Baseline demographic and clinical characteristics of study patients

Parameter	Drug group (Frankincense) (n = 33)	Control group (n = 37)	*P-value*
Age (years; mean ± SD)	59.82 ± 9.00	57.32 ± 8.18	0.229
Sex (n)			
Male	4 (12.1%)	4 (10.8%)	1.000
Female	29 (87.9%)	33 (89.2%)	
Duration of OA (month; mean [IQR])	54 [30–87]	36 [12–87]	0.158
Comorbidity (n)			
Diabetes	1 (3.0%)	1 (2.7%)	
Hypertension	2 (6.1%)	4 (10.8%)	
Dyslipidemia	3 (9.1%)	1 (2.7%)	
Hypothyroidism	0 (0.00%)	3 (8.1%)	
Hyperthyroidism	1 (3.0%)	0 (0.00%)	
Migraine	2 (6.1%)	1 (2.7%)	
Hypertension + Dyslipidemia	2 (6.1%)	2 (5.4%)	
Diabetes + Dyslipidemia	1 (3.0%)	1 (2.7%)	
Hypothyroidism + Hypertension	1 (3.0%)	0 (0.00%)	
Hypothyroidism + Diabetes	0 (0.00%)	1 (2.7%)	
Hypothyroidism + Diabetes + Dyslipidemia	0 (0.00%)	2 (5.4%)	0.469
Hypothyroidism + Diabetes + Dyslipidemia + Hypertension	0 (0.00%)	1 (2.7%)	
Concurrent drugs (n)			
Metformin	0 (0.00%)	1 (2.7%)	
Losartan	1 (3.0%)	1 (2.7%)	
Valsartan	0 (0.00%)	1 (2.7%)	
Aspirin	0 (0.00%)	1 (2.7%)	
Atorvastatin	3 (9.1%)	0 (0.00%)	
Levothyroxine	0 (0.00%)	2 (5.4%)	
Amlodipine	0 (0.00%)	1 (2.7%)	
Metformin + Losartan + Atorvastatin + Pantoprazole + Propranolol	2 (6.1%)	1 (2.7%)	
Losartan + Furosemide + Atorvastatin + Aspirin	1 (3.0%)	0 (0.00%)	
Metformin + Rosuvastatin + Aspirin	1 (3.0%)	0 (0.00%)	0.367
Losartan + Aspirin	1 (3.0%)	1 (2.7%)	
Losartan + Atorvastatin + Aspirin	1 (3.0%)	0 (0.00%)	
Atorvastatin + Aspirin	0 (0.00%)	1 (2.7%)	
Metformin + Glibenclamide	1 (3.0%)	0 (0.00%)	
Losartan + Atorvastatin	0 (0.00%)	2 (5.4%)	
Metformin + Atorvastatin + Levothyroxine	0 (0.00%)	1 (2.7%)	

### Effectiveness evaluation

As shown in Table 2, for all evaluated outcome variables (total WOMAC score as well as its subscales, VAS score, and PGA score), there was a significant decrease from baseline in both groups (*P* < 0.001 for all). Furthermore, in contrast to the baseline values, the end-of-intervention values for all parameters were significantly lower in drug group than placebo group (*P* < 0.001 for all), showing more effectiveness of drug compared to placebo.

**Table 2:** Pre- and post-intervention values of parameters and their comparison between the two groups

Parameters	Time	Group		*P value*	Mean difference^*^ (95% CI)
		Drug	Placebo		
Pain severity	Baseline (week 0) End (week 4) *P-value*	16 [13–18] 4 [2–7.5] < 0.001c	15 [10–19] 8 [4–17.5] < 0.001c	0.958_a_ 0.001_b_	3.91 ± 1.44 (1.02–6.80)
Flexibility	Baseline (week 0) End (week 4) *P-value*	6 [4–7] 2 [0–2] < 0.001c	6 [4–8] 4 [1.5–6] < 0.001c	0.356_a_ 0.001_b_	1.84 ± 0.50 (0.83–2.85)
Function	Baseline (week 0) End (week 4) *P-value*	46.54 ± 11.24 22.30 ± 15.48 < 0.001d	47.68 ± 11.55 36.00 ± 18.40 < 0.001d	0.68_b_ < 0.001_b_	13.70 ± 4.09 (5.53–21.86)
Total score of WOMAC	Baseline (week 0) End (week 4) *P-value*	67 [58–79] 24 [15–36.5] < 0.001c	70 [54–83.5] 52 [25–74] < 0.001c	0.646_a_ 0.001_b_	19.08 ± 5.71 (7.67–30.48)
VAS score	Baseline (week 0) End (week 4) *P-value*	9 [7–10] 4 [2.5–5] < 0.001c	9 [8–9.5] 6 [3.5–9] < 0.001c	0.804_a_ 0.001_b_	1.92 ± 0.62 (0.67–3.16)
PGA score	Baseline (week 0) End (week 4) *P-value*	3 [2.5–4] 1.8 [1–2.5] < 0.001c	3.5 [3, 4] 3 [1.9–3.5] < 0.001c	0.918_a_ 0.001_b_	0.78 ± 0.26 (0.26–1.30)

*WOMAC* Western Ontario and McMaster Universities Osteoarthritis Index, *VAS* visual analogue scale, *PGA* patient global assessment

^a^Mann-Whithney U test

^b^ANCOVA test

^c^Wilcoxon Signed Rank test

^d^Paired Sample T-test

^*^Mean ± SE of difference for end values according to ANCOVA test

### Side effects

Only one person in placebo group complained of itching and redness at the application site at the first days; so, she was excluded from the research. No other patient reported any adverse effect during the intervention.

## Discussion

In this study, topical solution of Frankincense extract could decrease pain severity and stiffness of knee and improve the patients’ daily activity.

To the best of our knowledge, this is the first work evaluating Boswellia extract as a topical form in OA patients. Previously, several animal and human studies have shown the beneficial effects of Boswellia in arthritis.

An animal study demonstrated that boswellic acid could significantly suppress the increased level of lysosomal β-glucuronidase and lactate dehydrogenase enzyme releasing from neutrophils and also decreased TNF-α level in gouty arthritic mice [19].

Several clinical trials showed the effectiveness of *B. serrata* on OA. In the study of Kimmatkar et al., the effects of this plant in the reduction of pain, improvement of patients’ function, and increase of walking distance and the ability of climbing the stairs were demonstrated [15]. In the study of Majeed et al., the oral use of *B. serrata* extract by OA patients could significantly decrease WOMAC and VAS scores compared to placebo at the end of 4th month and also improved articular cartilage destruction based on radiography assessment [16]. However, the results of our four-week trial showed the positive effects of the extract even at a shorter duration. Similar results have been obtained in other trials of *B. serrata* extract in OA patients [20–27] which are consistent with ours. Of note, all of these works have evaluated oral form of the extract, while we applied the topical form. Therefore, it seems that topical use of frankincense extract can improve OA in terms of pain severity and joint flexibility, as well as patients’ quality of life (as determined by PGA score).

The biological effects of Boswellia are mainly related to boswellic acids, of which AKBA is the most important one. This compound demonstrates anti-inflammatory and anticancer effects by selective and non-competitive inhibition of 5-lipoxygenase in the pathway of leukotriene biosynthesis [11, 22, 25] and suppresses the expression and activity of matrix 3, 10 and 12 metalloproteinases in human capillary endothelial cells [28].

Since topical agents have a role in the management of mild OA due to lower systemic side effects, topical frankincense could be an additional therapeutic option for this disorder as its tolerability seems to be better than topical NSAIDs and capsaicin.

## Conclusions

Topical oily solution containing enriched extract of boswellic acids could decrease pain severity and improve the function in patients with knee OA. However, as the first clinical study showing the positive effects of topical frankincense in OA patients, this work could be a basis for future larger trials to introduce an effective and safe herbal treatment for these patients.

## Limitations

The main limitations of our study were small sample size due to the occurrence of COVID-19 pandemic and short duration of intervention. Also, due to topical consumption, it is possible that the patients have taken various amounts of drug and placebo.

## Data Availability

The datasets used and/or analyzed during the current study available from the corresponding author on reasonable request.

## Abbreviations

OA: Osteoarthritis
NSAIDs: Non-steroidal anti-inflammatory drugs
AKBA: 3-O-acetyl-11-keto-β-boswellic acid
NF-ĸB: Nuclear factor kappa B
TNF-α: Tumor necrosis factor α
VAS: Visual analogue scale
WOMAC: Western ontario and mcmaster universities osteoarthritis index
PGA: Patient global assessment
